# The Painless Removal of Living Pulps

**Published:** 1885-04

**Authors:** G. M. Merritt

**Affiliations:** Jersey City


### THE PAINLESS REMOVAL OF LIVING PULPS.

BY DR. G. M. MERRITT, JERSEY CITY.

As it is often necessary to remove a living pulp, especially in mounting artificial crowns, it is very necessary for the comfort of the operator as well as the patient, that it should be done in a thorough yet painless manner, and often, if not always, at the first sitting. My method is as follows : we will suppose that a superior central incisor has the crown decayed and broken away, yet possessed of a live, healthy pulp, well covered with dentine. With a stump corundum, while kept wet, I grind the end of the root down until the patient manifests a disposition to flinch, owing to the close proximity of the stone to the pulp. I then put a Morey nerve canal drill in the hand piece, and keep it in readiness. Saturating a pledget of cotton with ether, and holding it on the end of the root and surrounding gum for one minute, I remove it and quickly place my drill in the center of the end of the root, and with a few quick turns of the engine I am able to penetrate quite to the apex without a particle of pain, or a word of remonstrance from the patient. This operation I have performed a great many times, and have never had but one patient say that it occasioned pain. In that exceptional case a lady was very anxious to know just what was to be done with each particular instrument, and I was foolish enough to explain each step in advance. She suffered more from apprehension than from actual pain, but her imagination made her think it hurt her. I have tried the knocking-out plan, described some time since by Dr. Mills, but have not had the success that I desired. I do not know that my plan is new, yet I have never seen an account of it reported in any of the journals, and as it has served me well I report it for the benefit of others.

